# Parliament and the Profession

**Published:** 1916-11-11

**Authors:** 


### PARLIAMENT AND THE PROFESSION.

### Picric Acid.

MR. BRACE, on behalf of the Home Office, informed Mr. Ellis Davies that the only diseases normally attributable to processes in the manufacture of, or involving the use of, picric acid are eczematous ulceration of the skin and poisoning by nitrous fumes, both of which are scheduled under the Workmen's Compensation Act.

### Food Supply at a Brighton Military Hospital.

MR. THOMAS-STANFORD asked the Secretary of State for War if he was aware that dissatisfaction exists among the wounded soldiers in the Royal Pavilion Hospital at Brighton as to the quality of the food supplied to them and the method of serving it. Mr. Lloyd George replied that he had heard of the dissatisfaction, and that an inquiry has been instituted into the matter.

### Wounded from Mesopotamia.

MR. CHAMBERLAIN, who was asked by Sir C. Kinloch-Cooke for information as to the alleged hardships and privations of the wounded in Mesopotamia, stated that the Viceroy of India was satisfied by an exhaustive inspection carried out by the G.O.C. in company with the Governor of Madras that there were nothing more than inconveniences of a temporary character, which were quickly dealt with. The Viceroy reported that there was no dearth of comforts or stores, and that private relief was merely supplementing official resources. In view of the sudden influx of patients there was a very slight deficiency in bedding and crockery, but at no time was there a lack of medicines. Any shortages in clothing and equipment were made good with all speed. Mr. Chamberlain said he had not sought to conceal any faults in administration where he believed them to exist, but he deprecated uncritical condemnation of the Indian Government and its officers, who felt deeply the aspersions cast upon them to which it is not open to them to reply; he was satisfied that there had been gross exaggerations as to the matter, and that the present position as to supplies is satisfactory.

### Venereal Diseases.

IN answering a question of Lord Winterton's in respect of venereal diseases, Mr. Bonar Law stated that the Home Secretary is considering legislation making criminal the wilful transmission of venereal disease by an infected to a healthy person. The Home Secretary also informed Colonel Griffiths that the subject of compulsory notification is under consideration in New South Wales and other Australian States.

